# Assessment of COVID-19 vaccination refusal among healthcare workers in Ethiopia

**DOI:** 10.3389/fpubh.2022.929754

**Published:** 2022-08-11

**Authors:** Elsa Tesfa Berhe, Adisu Tafari Shama, Mohammed Musa Ahmed, Hailay Abrha Gesesew, Paul R. Ward, Teferi Gebru Gebremeskel

**Affiliations:** ^1^Department of Reproductive Health, College of Health Sciences, Aksum University, Aksum, Ethiopia; ^2^Department of Public Health, Health Institute, Wollega University, Nekemte, Ethiopia; ^3^Benishangul USAID-THDR Project Officer, Assosa, Ethiopia; ^4^Centre for Research on Health Policy, Torrens University, Adelaide, SA, Australia; ^5^Department of Epidemiology, College of Health Sciences, Mekele University, Mekele, Ethiopia; ^6^Discipline of Public Health, Flinders University, Adelaide, SA, Australia

**Keywords:** refusal, COVID-19, vaccination, health and care workers, Ethiopia

## Abstract

**Background:**

Even though the emergence of the coronavirus disease 2019 (COVID-19) vaccine and the increasing vaccination rates are promising, there are reports of refusal to get vaccinated in a different segment of the population, including health care workers.

**Objective:**

This study determines the acceptance/refusal of the COVID-19 vaccination and its predictors among health care workers in Ethiopia.

**Methods:**

A cross-sectional survey was conducted among a group of health care workers at different health facilities in Ethiopia. Data were collected from 1 to 30 July 2020. Data were collected from 403 participants through an online Google submission form. Data were entered into Epi-info 7 and exported to SPSS version 20 for analysis.

**Results:**

Approximately 38.5% of the health care workers in the study had refused COVID-19 vaccination. Younger age groups vs. 40 and above years (age 20–24 year [AOR: 0.03, 95% CI (0.00, 0.48)], age 25–29 year [AOR: 0.02, 95% CI (0.00, 0.49)], and age 30–34 year [AOR: 0.04 (0.00, 0.49)] and medical doctors vs. Nurses [AOR: 0.06, 95% CI, (0.01, 0.42)] were reported as risk factors; also, academic working staff vs. hospital staff [AOR: 4.42, 95% CI (1.85, 10.54)] was reported as a protective factor toward refusal of COVID-19 vaccination.

**Conclusion:**

Two-fifths of health care workers in Ethiopia were indicated to refuse COVID-19 vaccination, implying a significant barrier to achieving WHO's a target of 70% double vaccination rate by mid-2022.

## Introduction

Coronavirus disease 2019 (COVID-19) is a pandemic and a current public health priority affecting the general population globally (1). Health care workers (HCWs) are being infected at higher rates than the general population due to the nature of their work (2, 3). Evidence shows that the introduction of the COVID-19 vaccine brought a substantial reduction in mortality and severe form of the disease (4). The acceptance rate of COVID-19 vaccination among health workers is higher than among non-health workers (5). The refusal level of COVID-19 vaccination among HCWs is variably reported from 23.1% in France (6), 60% in Hong Kong (7), 61% in Ghana (8), 61.7% in Nepal (9), 62.3% in the Democratic Republic of the Congo, (10), and 9.9% in South Africa (11). Evidence also shows that more than 80% of the 22 countries with the highest-income have fully vaccinated their workers, but less than one in ten people are fully vaccinated in Africa (12).

Studies in the United States reported lower COVID-19 vaccination refusal in physicians vs. nurses (13), as well as socio-demographic factors related to age group and workplaces of COVID-19 vaccination (6, 14–16). As COVID-19 vaccines continue to be distributed and administered in many countries, including Ethiopia, refusal toward the vaccine is becoming a challenge and a barrier to cover a large proportion of the vulnerable population. In fact, the WHO has identified vaccine hesitancy as one of the top ten threats to global health in 2019 (17).

The refusal rate of COVID-19 vaccination in Africa is not studied very well, and there was not much work done in Ethiopia on the issue. The findings from these professionals would help policy makers in the health sector to improve vaccine acceptance, which would contribute to the control of COVID-19 pandemics and essentially facilitates meeting the WHO targets to vaccinate 70% by mid-2022. This study aims to report the refusal rate of COVID-19 vaccination and associated factors among HCWs in Ethiopia.

## Methods

### Study design, setting, and population

An online cross-sectional survey was conducted among a group of HCWs at different health facilities in Ethiopia, and data were collected from 1 to 30 July 2020. Ethiopia is the second-most populous country in Africa with an estimated population of more than 110 million. Ethiopia has 10 regional states including Afar, Amhara, Benishangul-Gumuz, Gambella, Harari, Oromia, Somali, Sidama, Southern Nations, and Nationalities and People (SNNP), and Tigray, and two chartered cities namely Addis Ababa and Dire Dawa. Data were collected from both chartered cities and all regional states except Tigray as the region is under defacto blockade and there is no network because of the 2020/2021 war. HCWs, including physicians, pharmacists, nurses, and laboratory technicians, who have access to submit a Google form were eligible to be included in the study. We have included HCWs of Ethiopian residents. Incomplete surveys were excluded from the analysis.

### Sample size and procedure

The sample size was estimated by using the Kish Leslie formula (1965).


$$\begin{array}{l} \text{Formula}:\text{n}={\left(\text{Z}\right)}^{2}\text{P}\left(1-\text{P}\right)/{\text{e}}^{2} \end{array}$$

Where,

*n* = the required minimum sample size

Z = constant standard normal de*via*te (1.96% confidence level)

P = estimated intentions of acceptability of vaccination against COVID-19 which is 50% (no previous study found in Ethiopia).

e = margin of error on p (set at 5%) and, assuming a 5% non-response rate, the actual sample size for this study was 403 HCWs. The study participants were selected using the snowball sampling technique through the authors' networks on the popular social Medias (Messenger/Facebook and Whatsapps).

### Variables, measurement, and data collection process

Academic staffs are staffs who work in higher health teaching institutions including staff personnel who hold an academic rank with titles such as professor, associate professor, assistant professor, instructor, lecturer, or the equivalent of any of these academic ranks. Health offices are the category of place of work for health workers who practice in offices such as district health offices, zonal health department, regional health bureaus, and federal ministry of health. HCWs ‘intention to accept the COVID-19 vaccine, the response variable, was assessed by one question asking “will you get a vaccine if it is available?” Vaccine refusal was assessed by this question proactively since there was no COVID-19 vaccination in Ethiopia at that time. Participants' responses were dichotomized into ”Yes” and "No.” We have also collected data of some independent variables such as age, sex, ethnicity, marital status, income, profession, and work areas. We used the online data collection method and we created a Google form for participants to invite, complete and submit the form. A questionnaire link was shared with recruited groups for HCWs on Messenger/Facebook and Whatsapps. Data were collected using a pretested self-administered questionnaire adapted from previous studies (10, 18). Standard structured questionnaires were then translated to working languages of each regional state by expertise with similar experience. In addition to this, the notification was written in the introduction part of the questionnaire for respondents to fill the questionnaire only once.

### Data analyses

Data were entered into Epi-info 7 and analyzed with SPSS version 20. We summarized data using descriptive statistics using frequencies, and percentages. Bivariate logistic regression was used to select candidate variables for multivariate binary logistic regression. Adjusted odds ratios (AOR) with corresponding 95% CI were calculated to assess the relationship between vaccine refusals and linked variables. Finally, the Hosmer and Lemeshow's test used the adequacy of the fitted model. In this study, on the final model, a *p*-value of <0.05 was assumed to be a statistically significant value.

## Results

In total, 403 health care workers were included in the study with 100% response rate as authors stopped sharing the google form link when the required sample size was reached. Mean age of the respondents was 29.1 ± 5.7 years. Most (66.5%) of the health care workers were married, followed protestant religion 239 (59.3%), and had completed BSc degree level of education 172 (42.7%). Also, 198(49.1) participants were public health professionals followed by 99 (24.6%) nurses. Table 1 describes the demographic characteristics of the study participants.

**Table 1:** Social-demographic Distribution of the Respondents (*N* = 403).

**Variables**	**Frequency**	**Percent (%)**	
Age	20–24	10	2.5
	25–29	168	41.7
	30–34	164	40.7
	35–39	40	9.9
	40 and above	21	5.2
Sex	Male	363	90.1
	Female	40	9.9
Resident	Urban	338	83.9
	Rural	65	16.1
Religion	Orthodox	90	22.3
	Protestant	239	59.3
	Muslim	49	12.2
	Waqefata and others^a^	25	6.2
Ethnicity	Oromo	372	92.3
	Amh2ara	12	3.0
	Guraghe & others^b^	19	4.7
Marital status	Single	135	33.5
	Married	268	66.5
Educational status	Diploma	23	5.7
	BSc Degree	172	42.7
	Master Degree	152	37.7
	Doctorate Degree and or above	56	13.9
Family size	<5	350	86.8
	>=5	53	13.2
Monthly income	1,651–3,200 Ethiopia birr	6	1.5
	3,201–5,250 Ethiopia birr	35	8.7
	5,251–7,800 Ethiopia birr	137	34.0
	7,801–10,900 Ethiopia birr	138	34.2
	>=1,0900 Ethiopia birr	87	21.6
Work experience in years	< =10	307	76.2
	>10	96	23.8
Place of work	Hospital	91	22.6
	Health center	94	23.3
	Academic staff	116	28.8
	Private	13	3.2
	Others^c^	13	3.2
	Health offices	16	18.9
Professional category	Nurse	99	24.6
	Midwifery	30	7.4
	Medical doctor	24	6.0
	Medical laboratory	7	1.7
	Pharmacy	18	4.5
	Psychiatry	6	1.5
	Environmental health	6	1.5
	Public health	198	49.1
	Others^d^	15	3.7

a *Hindu, Apostolic Christian;*

b *Burjii, Sidama, India;*

c *Non-Governmental Organization, Blood bank;*

d *Integrated Emergency surgery and Obstetric (IESO), Health Education and Anesthesia*.

### Prevalence of COVID-19 vaccination refusal rate and associated factors

Two out of five (38.5%) health care workers refused COVID-19 vaccination in Ethiopia. Age, religion, ethnicity, marital status, educational status, family size, work experiences, place of work, professional category were included in the bivariate binary logistic regression analysis. In the multivariable logistic regression analysis, age, place of work, professional category were significantly statistically associated with COVID-19 vaccine refusal. The odd of reporting a refusal to COVID-19 vaccination was lower among medical doctors compared to nurses [AOR: 0.06, 95% CI, (0.01, 0.42)]. Academic staffs were four times [AOR: 4.42, 95% CI (1.85, 10.54)] more likely to refuse to COVID-19 vaccination than the hospital staffs. The odds of reporting a refusal to the COVID-19 vaccination were lower among younger age groups compared to 40 and above years (Table 2).

**Table 2:** Socio-demographic factor associated with COVID-19 vaccine refusal among HCWs.

**Variables**		**COVID-19 Refusal**	**Vaccine**	**OR [95% CI]**		***P*** **-value**
		**Refused (%)**	**Not Refused (%)**	**COR**	**AOR**	
Age	20–24	5 (2.33)	5 (2.66)	0.31(0.00, 0.56)	0.03 (0.00, 0.48)^*^	0.014
	25–29	90 (41.9)	78 (41.5)	0.36 (0.1, 0.49)	0.02 (0.00, 0.20)^*^	0.001
	30–34	99 (46)	65(34.6)	0.48 (0.01, 0.68)	0.04 (0.00, 0.49)^*^	0.012
	35–39	5 (2.33)	35 (18.6)	0.05 (0.01, 3.21)	0.23 (0.02, 3.37)	0.282
	40 and above	16 (7.44)	5 (2.66)	1	1	1
Religion	Orthodox	63 (25.4)	27 (17.4)	1	1	1
	Protestant	124 (50.0)	115(74.2)	0.46 (0.28, 0.78)	0.27 (0.14, 0.54)	0.000
	Muslim	40 (16.1)	9 (5.8)	1.91 (0.81, 4.47)	2.61 (0.93, 7.34)	0.070
	Waqefata and others^a^	20 (8.5)	5 (2.6)	1.71 (0.71, 7.18)	1.47(0.38,5.73)	0.577
Ethnicity	Oromo	223 (92.3)	143 (92.3)	0.50 (0.09, 1.05)	3.22 (0.65, 16.0)	0.152
	Amhara	5 (1.2)	7 (5.8%)	0.26 (0.01, 0.38)^*^	0.12 (0.01,1.05)	0.056
	Guraghe & others^b^	14 (6.5)	5 (1.9%)	1	1	1
Marital status	Single	81 (32.7)	54 (34.8)	1	1	1
	Married	167 (67.3)	101 (65.2)	1.10 (0.72, 1.68)	1.36 (0.28,5.85)	0.321
Educational status	Diploma	11 (4.4)	12 (7.7)	1	1	1
	BSc degree	95 (38.3)	77 (49.7)	1.35 (0.56, 3.22)	0.69 (0.22,2.19)	0.523
	Master degree	97 (39.1)	55 (35.5)	1.92 (0.79, 4.65)	1.30 (0.36, 4.69)	0.686
	Doctorate and or above	45 (18.1)	11 (7.1)	4.46(1.56, 12.8)	1.62 (0.34, 7.74)	0.544
Family size	<5	218 (62.3)	132 (37.7)	1	1	1
	>=5	30 (56.6)	23 (43.4)	0.79 (0.44, 1.42)	0.74 (0.32, 1.25	0.524
Work experience	< =10	198 (79.8)	109 (70.3)	1	1	1
	>10	50 (20.2)	46 (29.7)	0.59 (0.38. 0.95)	0.31 (0.17,0.58)	0.000^*^
Place of work	Hospital staff	50 (20.2)	41 (26.5)	1	1	1
	Health center	53 (21.4)	41 (26.5)	1.06 (0.59, 1.89)	1.85(0.82, 4.19)	0.140
	Academic staff	83 (33.5)	33 (21.3)	2.06 (1.16, 3.67)	4.42(1.85,10.54)^*^	0.001^*^
	Private	8 (3.6)	5 (2.6)	1.85 (0.53, 6.43)	1.89 (0.43,8.47)	0.402
	Others^c^	7 (2.8)	6 (3.9)	0.96 (0.29, 3.07)	1.29 (0.29,5.75)	0.738
	Health offices	46 (18.5)	30 (19.4)	1.26 (0.68, 2.33)	2.34 (0.94, 5.85)	0.069
Professional category	Nurse	69 (27.7)	30 (19.4)	1	1	1
	Midwifery	15 (6.0)	15 (9.7)	0.435 (0.19, 1.00)	1.12(0.42,3.0)	0.820
	Medical laboratory	19 (7.63)	5 (3.2)	2.174(0.68, 6.9)	3.68(0.63, 21.7)	0.004^*^
	Medical doctor	5 (2.0)	8 (5.2)	0.27 (0.03, 0.94)	0.06(0.01, 0.4)^*^	0.15
	Pharmacy	10 (4.0)	8 (5.2)	0.54 (0.19, 1.51)	0.39 (0.09,1.62)	0.197
	Environmental health	5 (2.0)	5 (3.2)	0.44 (0.08, 2.28)	0.04(0.00, 0.7)^*^	0.023^*^
	Public health	116(46.6)	78 (51)	0.64 (0.38, 1.07)	1.21(0.29, 5.1)	0.792
	Others^d^	10 (4.07)	5 (3.2)	0.87(0.27, 2.76)	0.48(0.23, 0.01)	0.053

a *Hindu, Appostolic Christian;*

b *Burjii, Sidama, India;*

c *Non-Governmental Organization, Blood bank;*

d *Integrated Emergency surgery and Obstetric (IESO), Health Education. COR, Crude odds ratio; AOR, Adjusted odds ratio*.

* *P-value < 0.05*.

## Discussion

This study was aimed to determine the health and care worker's rate of COVID-19 vaccine refusal in Ethiopia. The finding showed that two-fifth of health care workers refused the COVID-19 vaccine. This finding is consistent with findings from other studies in the United States (35%) (19) and Jordan (36.3%) (20). The refusal levels of the COVID-19 vaccine in our study was lower than those of the cross-sectional study conducted in southwestern Ethiopia (52.3%) (21), Hong Kong (60%) (7), Ghana (61%) (8), Nepal (61.7%) (9), and the Democratic Republic of the Congo (62.3%) (10); and higher than France (23.1%) (6), United States (27%) (22), Italy (13.9%) (23), and South Africa (9.9%) (11). The difference in magnitude could be due to the different timings of the introduction of the vaccine, the nature of data collection methods, level of miss- and disinformation about the safety of the COVID-19 vaccine. The current finding on the COVID-19 vaccination refusal in Ethiopia is a considerable high number and needs attention.

Hence, building trust regarding the ability of the government and other concerned bodies is crucial. Thus, to increase vaccine uptake and acceptance, the Ethiopian Ministry of Health and other concerned bodies should build strategies such as organize intercultural health advocating sessions for HCWs and the community, increasing knowledge and skill of health advocators in terms of vaccine information and interpersonal communication, engage key community leaders in the information provision, engage vaccine users (HCWs and the community) in providing agreed vaccination information and make informed decisions, engage HCWs in an empathic way and design different vaccine related information communication platforms (24).

In this study, some socio demographic variables predicted the level of refusal to the COVID-19 vaccination in Ethiopia. The discrepancies in values of some variables were found particularly for variables like professional category (49.1% public health), ethnicity (92.3% Oromo), and religion (59.3% protestant). This may attribute to the non-random sampling technique as well as the absence of some regions from the study. Nurses were more likely to refuse to get vaccinated to COVID-19 than medical doctors, a finding consistent with findings from the United States (13). This may be due to the fact that the level of misinformation or disinformation toward the vaccine may be higher among nurses compared to medical doctors. Given nurses are the front-line workers in many departments of health facilities, this finding is extremely concerning. It is important to explore the potential reasons for refusal qualitatively and quantitatively. Academic staffs were four times more likely to refuse to get the COVID-19 vaccine than clinical staff, which is similar to a study conducted in California where higher proportions of administrative, non-clinical staff HCWs were among the vaccine-hesitant and refuses (16). This may be due to the fact that clinicians may be assumed that they are at risk of infection, severity, and morbidity than non-clinicians. This study showed that younger age groups were less likely to refuse the COVID-19 vaccine, a finding similar to the studies conducted in France (25), and the United Kingdom (26) but inconsistent with a study conducted in Saudi Arabia (14), the US (27), and Greece (15). This could be owing to the active engagement of young HCWs in various social media platforms, which are mostly disseminating right information from reliable sources.

In our study most (90.1%) of the health care workers were male, urban residence (83.9%), and had ethnic Oromo (92.3%). Surprisingly ethnicity, residence and gender were not an independent predictor for COVID-19 vaccination refusal in our setting. In this study ethnicity, residence and gender did not have a statistically significant association with the COVID-19 vaccination refusal but studies conducted in resident Ohio, Latin America and the Caribbean reviled that non-Hispanic black, female gender and rural resident a predictor for COVID-19 vaccination refusal (28, 29). This is probably may be due to the different timings of the introduction of the vaccine, small sample size and the nature of the study design, level of miss- and disinformation about the COVID-19 vaccine.

The study has the following limitations. First, findings from a cross-sectional study design could not confirm the cause and- effect relationship. Second, since the study was an online survey and self-reporting some sections of participants such as those who did not access the internet or were less likely to use the internet may be denied. We have already highlighted that, participants from the entire Tigray are not included in the study due to the total shutdown of the internet. Furthermore, the small sample size may affect the precision of the study. Third, due to the nature of the study design, we could not understand the in-depth reasons behind the refusal among the aforementioned groups. Forth, written instruction was the only mechanism used to avoid repetitive filling of the questionnaire. Finally, the distribution of health professionals in this study may vary from the actual distribution of the professional category in Ethiopia.

## Conclusion

A considerable percentage of health care workers in Ethiopia (38.5%) indicated a refusal to the COVID-19 vaccination. Health care workers of younger age groups, clinicians, and professional categories were highly likely to refuse to get vaccinated. These imply the need to target these sections of the population, and the need to heed to address and understand the refusal of COVID-19 vaccination among health workers. Moreover, the issue of refusal among health workers may also affect the general population by implication and hence, it requires serious attention. We recommend for researchers to conduct a qualitative study for an in-depth understanding of potential barriers to refusal, and also perform large-scale surveys by including additional variables.

## Data availability statement

The raw data supporting the conclusions of this article will be made available by the authors, without undue reservation.

## Ethics statement

The studies involving human participants were reviewed and approved by Ethical clearance was obtained from the Institutional Review Committee of the College of Medicine and Health Sciences, Aksum University. Study participants were also given a full right to refuse/withdraw from the study process at any time in the study process. Participants in the study were informed about the purpose of the study and the privacy of information provided. Written informed consent for participation was not required for this study in accordance with the national legislation and the institutional requirements.

## Author contributions

ETB, AS, and TG designed the study and performed the statistical analysis, drafted the paper, and data analysis. MA, HG, and PW participated in paper writing. All authors read and approved the final paper.

## Conflict of interest

The authors declare that the research was conducted in the absence of any commercial or financial relationships that could be construed as a potential conflict of interest.

## Publisher's note

All claims expressed in this article are solely those of the authors and do not necessarily represent those of their affiliated organizations, or those of the publisher, the editors and the reviewers. Any product that may be evaluated in this article, or claim that may be made by its manufacturer, is not guaranteed or endorsed by the publisher.

## Abbreviations

CDC: Centre of Disease Control
COVID-19: Coronavirus Disease-2019
MERS: Middle East Respiratory Syndrome
SARS: Severe Acute Respiratory Syndrome
SARS-COV2: Severe Acute Respiratory Syndrome Corona Virus 2
WHO: World Health Organization
CI: Confidence Intervals
OR: Odds Ratio
HCW: Healthcare Worker.
